# Making social robots adaptable and to some extent educable by a marketplace for the selection and adjustment of different interaction characters living inside a single robot

**DOI:** 10.3389/frobt.2025.1534346

**Published:** 2025-04-08

**Authors:** Sebastian Reitelshöfer, Nina Merz, Gabriela Garcia, Yuqiang Wei, Jörg Franke

**Affiliations:** Institute for Factory Automation and Production Systems, Friedrich-Alexander-Universität Erlangen-Nürnberg, Erlangen, Germany

**Keywords:** social robots, software architecture, marketplace, social skills, adaptability, robot operating system ROS

## Abstract

The increasing integration of autonomous robotic systems across various industries necessitates adaptable social interaction capabilities. This paper presents a novel software architecture for socially adaptable robots, emphasizing simplicity, domain independence, and user influence on robotic behaviour. The architecture leverages a marketplace-based agent selection system to dynamically adapt social interaction patterns to diverse users and scenarios. Implemented using ROS2, the framework comprises four core components: scene analysis, a bidding platform, social agents, and a feedback service. A Validation through simulated experiments shows the architecture’s feasibility and adaptability, with respect to varying feedback conditions and learning rates. This work lays the foundation for scalable, adaptable, and user-friendly robotic systems, addressing key challenges in industrial and social robotics. Future improvements include enhanced scene analysis, integration of machine learning techniques, and support for more complex behavioural scripts.

## 1 Introduction

Foreseeable, a growing number of autonomous robotic systems will be employed in numerous contexts including healthcare, service and consumer robotics but also in production and logistics with more complex and location independent applications. Aside from advanced mechatronic developments, recently evolved AI techniques like large language models (LLM) and vision language models (VLM) are key enablers in this respect. Reinforcement learning, task planning or decision making (Jeong et al., 2024) is fuelling those complex autonomous robotic applications. Becoming progressively more autonomous, it is highly likely that robotic systems will more often get into close proximity and contact with very different groups of people. For example, a future mobile service robot with enhanced capabilities may not only execute intralogistics tasks on a confined shopfloor but also manoeuvre between different buildings and across company premises. As a result, it will likely encounter not only trained shopfloor workers but also security staff, personnel from other companies, and visitors. Realizing accepted interaction or at least pleasant active coexistence with a broad spectrum of different user categories will need basic social adaptability of robotic systems. Of course, if the tasks get more in the direction of social interaction at the core of a specific application, social skills of robotic systems become correspondingly even more important. This relevance is in addition underlined by the World Economic Forum, stating that social robotics is one of the most important emerging technologies (Corinna and Lathan, 2019).

Among other technological components, a software architecture to realize and frame social adaptability is one core element to realize social robots. Architectures for social robotic systems have already been proposed in publications from the last century (O'Hare et al., 1999). A frequently described application scenario for deriving software architectures for social robots addresses the interaction with older persons. For example, Bonaccorsi et al. describe various cloud-based services with relevant functions for service robots for elderly people (Bonaccorsi et al., 2016). Another architecture consisting of the four components navigation, person tracking, localisation and interaction that is designed for a specific service robot operating as an assistant for older people has been implemented by Coşar et al. (2020). A service-based architecture with over 30 subcomponents is presented for application scenarios of social robots in geriatric care (Portugal et al., 2019). A system for carrying out exercises with older people, which uses six modules to record people’s condition, trigger an exercise pattern and give instructions via text-to-speech, is presented by Fasola and Mataric (2013). Fan et al. present a robot coach architecture for elderly care based on multi-user engagement models (Fan et al., 2017). In another approach, a rule engine is used to execute certain robotic actions on a NAO robot based on recorded medical data of older people and a knowledge database to trigger suggestions by the robotic system (Torta et al., 2014). Gross et al. describe a layer-based architecture for a specific robot for interacting with elderly people with five layers and over 20 subcomponents (Gross et al., 2011).

The therapeutic area is another setting for which several different architectures for social robots have already been developed. An exemplary architecture describes the behaviour of a robot on an abstract level and then maps it onto the morphology of the robot (Cao et al., 2016). Cao et al. also present a more complex architecture with over 20 subcomponents for the therapeutic context (Cao et al., 2019). There are systems that are specifically designed for screening people with autism spectrum disorder using for instance a special bird-like robot (Dehkordi et al., 2015). For the training children with autism spectrum disorder another approach exists with a straight forward ROS architecture where a therapist can select behaviours on the robot Pepper (Sessner et al., 2022). An example of a more complex, level-based architecture for the implementation of social robot applications in the medical field and for healthcare is presented for the robotics middleware ROCOS (Jayawardena et al., 2016). Another publication shows a proposed architecture for an assistance system in cardio-rehab scenarios for robots using the Naoqi framework, based on state machines (Casas et al., 2018).

In addition to the specific fields of application addressed in the medical context, some architectures for different other contexts have also been developed. Kim et al. show a very complex level-based architecture in which social robots with potentially many possible applications are orchestrated as agents (Kim et al., 2009). Furthermore, an approach to accompany users’ planned activities throughout the day and to trigger adapted behaviour patterns with a service robot is described by Louie et al. (2014). Another publication presents a set of software modules for using a specific robot platform for games with humans (Gonzalez-Pacheco et al., 2011). Yet another quite complex architecture with 15 components for the implementation of social robots in different application scenarios based on layers is presented by Asprino et al. (2022). An emotion-based approach for social robots in general, which triggers certain behaviour patterns based on detected emotions on the basis of a complex hierarchical behaviour control, is described by Hirth et al. (2011). A multimodal, emotion-based approach for human robot interaction is presented by Hong et al. (2021), whereby four main components encapsulate a quite complex architecture with a total of 18 elements. Social navigation, which has already been considered in numerous publications (Mavrogiannis et al., 2023), can also be accounted as a relevant field of technical implementations for social robots. Bera et al. present a multimodal emotion learning approach for socially assistive robot navigation (Bera et al., 2020). Multi-agent systems are described as well in the context of social navigation. Similar to the work of Kim et al. mentioned at the beginning of this section, a multi-agent system for path planning of social robots is described by Chandra et al. (2023). Specialized simulators can be used to carry out studies on social navigation with multi-agent systems (Chandra et al., 2024). Independent of the specific application, like solving navigational tasks, multi-agent systems are often used to describe approaches where several robots are coordinated, as shown for example in a case study (Botelho et al., 2020). In addition, multi-agent systems can also be used, for example, to enable different robots to share the results of learning approaches (Orr and Dutta, 2023). The area of socially assistive robotics, which is characterized by social rather than physical support of humans, is also detached from a specific application (Feil-Seifer and Mataric, 2005). An generalist approach to the behavioral control combing a ontology with artificial intelligence that conceives socially assistive robots as complex systems with a multitude of internal and external elements is presented by Umbrico et al. (2020).

To summarise, the brief overview provided shows that a large number of proposed architectures in the context of social robots is primarily aimed at geriatric or medical applications. If approaches contain multi-agent concepts, several robots are practically always mapped as agents and not several agents are assumed to live in one single robot. Just a view architectural concepts are addressing application domains other than health and care like for example education or are application agnostic. Social navigation to some extends stands out among these fields of application, as this area is being researched relatively intensive. In the class of socially assistive robots, domain-independent approaches are also being developed. In future, there may be further approaches to control socially assistive robots in multi domain scenarios in conjunction with artificial intelligence. To date, no architectures for social robots have been proposed directly and specifically for the use in an industrial production context. Also all the described approaches are somewhat intended to realize complex behavior patterns at the cost of high complexity rather than allowing for simple adaptation mechanisms to different user groups. Furthermore, in most cases a stringent development for the specific robotic system is recognisable, which was the object of investigation in the respective projects. There continues to be a need for research into software systems that are designed to develop adaptable social robots as independently as possible of the application domain and specific systems. In addition, many of the architectures are very complex, which, together with the aforementioned special development for certain target systems, makes it difficult for new developers to reuse those approaches for other projects.

## 2 Materials and methods

In the mentioned context, the use of a widespread framework such as the Robot Operating System (ROS) to implement the framework of an architecture for adaptable robots would also be desirable. ROS2 will enable the development of commercial ROS-based products and services on a broader scale in the future. Within the ROS ecosystem, packages or collections of packages have been established for a number of key issues in robotics, such as coordinate transformations (Foote, 2013), path planning (Coleman et al., 2014) and navigation (Macenski et al., 2020). Packages that specifically and comprehensively address sociality functionalities are not yet available.

From the short reflection of the state of the art and the summarised challenges described above, we derive five long-term development goals for our architecture proposal presented below, which should serve as guidelines for initial implementation and continuous further development.

- A software architecture for customisable social robots should be as domain-independent as possible.
- The basic structure of an architecture should be as easy to understand as possible in order to minimise the time required to familiarise users with its use.
- Humans should be able to understand, why a social robot selected to show a certain behaviour pattern and human end users should be able to influence robotic behaviour schemes.
- The concepts developed should be reusable.
- The architecture should be based on ROS.

**FIGURE 1:**
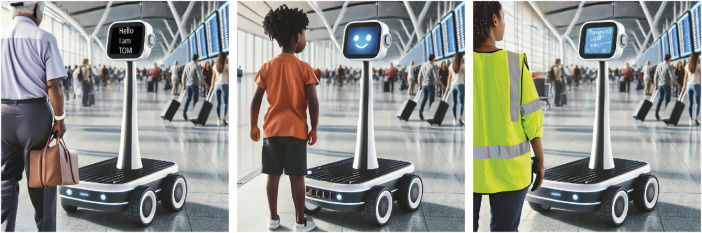
An adaptive social robot should be able to autonomously select different and best suited interaction schemes depending on the type of person an interaction takes place with. Based on user feedback it should also be able to automatically improve its selection rules. Images generated by the authors with DALL-E.

In the following section, we present a new software architecture for adaptable social robots. An overarching goal is to take into account the five guidelines mentioned above. The architectures described in the state of the art can certainly be used to map very complex social behaviour patterns for robotic systems - but often at the price of a high complexity of the underlying architectures. There is also a broad and heterogeneous state of the art for defining social robotics (Merz et al., 2024). In the following, very simple adaptation processes will initially be assumed as social adaptability by the authors. This selection is motivated by the fact that in robotic systems already in productive use, existing interaction patterns are practically always fixed and linearly scripted without any adaptability at all. For example, mobile transport robots available on the market for intralogistics tasks from different manufacturers can ask to clear a path via voice output. If they encounter an obstacle on their planned path, they repeat this request at a set time interval, depending on the setting, even if the path is blocked by a non-human obstacle. By constantly repeating these acoustic signals, nearby personnel can be significantly hindered in the fulfilment of their tasks. It can therefore happen that the positive feature of voice output is completely deactivated. In such cases, even a very simple and basic adaptability, such as changing the behaviour after a maximum number of unsuccessful communication attempts or selective communication based on object and person recognition, would very likely lead to a significant increase in acceptance. Asking a ladder to clear the way is useless even in the first attempt. The authors’ initial aim is to develop and research the architecture described below with regard to such simple adaption processes. A possible first application scope for categorisation is shown in Figure 1. In such a randomly selected example, there are several transport robot units at an airport. When an airport employee approaches such a system to have his or her tools or materials transported, an efficient interface designed for professional users is shown. The robot signals that it is ready for input with a green status light. The employee can then directly command a transport destination, the robot briefly displays the destination for confirmation and then drives off immediately. If, in this hypothetical example, such a fleet of transport systems is to be used simultaneously by passengers for transporting luggage who may never have had contact with a robot system before, the same transport robot must certainly offer a different interaction pattern with explanatory elements for such a user group. The constant initial presentation of information on how to use the robot would in turn limit the acceptance of the professional users mentioned above. It may even make sense to offer different interaction patterns for older and younger passengers. The aim of the architecture presented below is to allow robots to adapt automatically in such scenarios and at the same time to continuously improve the strategies for selecting suitable interaction patterns during operation based on user feedback.

### 2.1 Marketplace-based robotic solutions as an archetype

To put the architecture presented in this contribution into context, related approaches from other domains are briefly described. An agent-based structure will be used to encapsulate different social behaviours and functions. A marketplace-based approach is then to be utilised for selecting the agents. Market-based approaches build on microeconomic principles can generally be used as a paradigm for the coordination and control of complex robotic systems (Zlot et al., 2002). By modelling robots or software components as self-interested agents that participate in market-like interactions such as auctions or negotiations, these approaches can achieve emergent coordination and adaptation without relying on centralised control or explicit pre-programming (Lagoudakis et al., 2005). To illustrate the concept of the developed architecture for socially adaptable robots, a quite similar agent-based approach from the field of intralogistics (Scholz et al., 2019) is briefly described below. The aim of the comparable approach is to replace a central control unit in a logistics system that allocates transport jobs to intralogistics robots according to a pre-programmed scheme. Instead, Scholz et al. show that a self-regulating system can be created if transport jobs are placed on a bidding platform in a decentralised manner. Transport robots with different capabilities and parameters like size, speed or cost can then submit bids to process the jobs. These bids are generated on the basis of parameters that describe the characteristics of the individual robots. A transport robot with the shortest transport time, for example, then wins the transport job and carries it out. Finally, the central element of this approach is that the robots receive an evaluation after each completed task, which the robots then use to adjust their parameters for future bid generation. If, for example, a robotic system often wins transport jobs because it offers short transport times that it is then unable to fulfil during execution, the robot receives negative ratings with regard to transport speed and lowers its internal speed parameter in response. As a result, the robot henceforth makes more conservative bids in terms of speed. Simulations show that intralogistics systems organised in this way autonomously adjust themselves to optimal parameters (Scholz, 2019). The approach described below, which is inspired by the structure shown, can be used to realise an adaptive software architecture for social robot systems.

### 2.2 Architectural concept of a marketplace-based system for adaptable social robots

**FIGURE 2:**
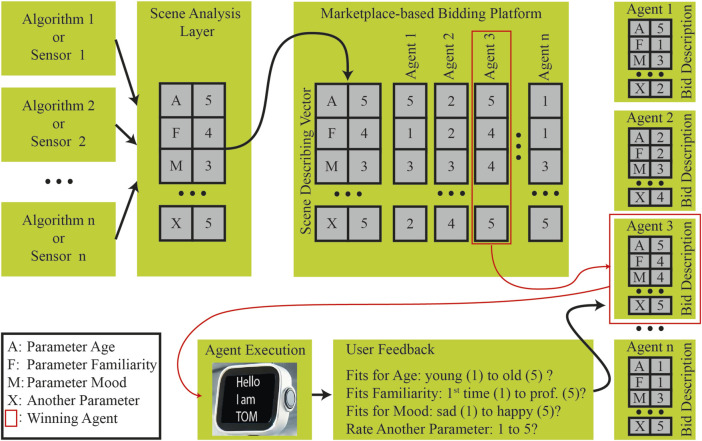
The proposed architecture includes four main components. The scene analysis maps an actual scene onto a set of parameters. This set is put on a market place. Agents can make bids to handle the scene. After winning and handling a scene an agent gets human feedback to adjust its parameters for future bidding rounds.

With respect to the example presented in Section 2.1 as an archetype, a marketplace-based architecture for social robots is proposed and described here. In this architecture, agents represent different social behaviour patterns for interacting with different user groups. This means that one single social robot is home to different agents. A scene analysis layer and a feedback system complete the four essential components of the first version of the architecture presented in Figure 2.

The functional principle of the architecture is shown in the following simplified form, inspired by the example in Section 2.1. The scene analysis module shown in Figure 2 on the left uses different sources of information like for example face recognition algorithms to map a current scene to a descriptive parameter set. This parameter vector describes selected aspects that characterise an upcoming interaction situation, whereby the individual parameters are mapped to numerical values. Possible parameters can be, for example, the age of an interaction partner, level of familiarity, mood or stress level. In such an example, the value of one for the “age” parameter can stand for a very young interaction partner and, for example, the value five for a very old person. The value one for the exemplary parameter “familiarity” represents an unknown first-time user and five means a previously known professional user and so on and so forth. After a scene analysis has been carried out, a created parameter set is placed as a new interaction task on the bidding platform of the marketplace module. All agents living in a robot can then place a bid for the processing of the upcoming scene. The bids represent the individual parameter sets that characterise the individual agents. For example, an agent can also have an “age” parameter, whose value of five indicates its suitability for interactions with very old people. Another parameter for “familiarity” with a value of two shows the suitability of the agent for rather inexperienced users. With the help of defined rules or other mechanisms such as learning approaches, the best offer or the best agent for handling the pending interaction is then selected by the marketplace module and scheduled for execution by this system. Initially, very simple agents are assumed, which either load an efficient professional-user interface or an interaction system for first-time users with explanatory videos before an actual command is accepted. Finally, at the end of such an interaction cycle, user feedback is obtained regarding all or selected parameters and then fed back to the agent that carried out the respective case. For example, if an agent receives feedback that it is well suited for elderly people, it will automatically increase the value of the “age” parameter for each such feedback according to defined rules. The parameters of agents on the system can thus be adjusted over several feedback cycles to influence the chances of their bids. User feedback can therefore be used to ensure that in similar situations, other or more appropriate agents win bidding rounds based on several feedback cycles, thereby automatically adapting the perceived behaviour of social robots over time to a certain extent.

### 2.3 ROS2 based implementation of the architecture

**FIGURE 3:**
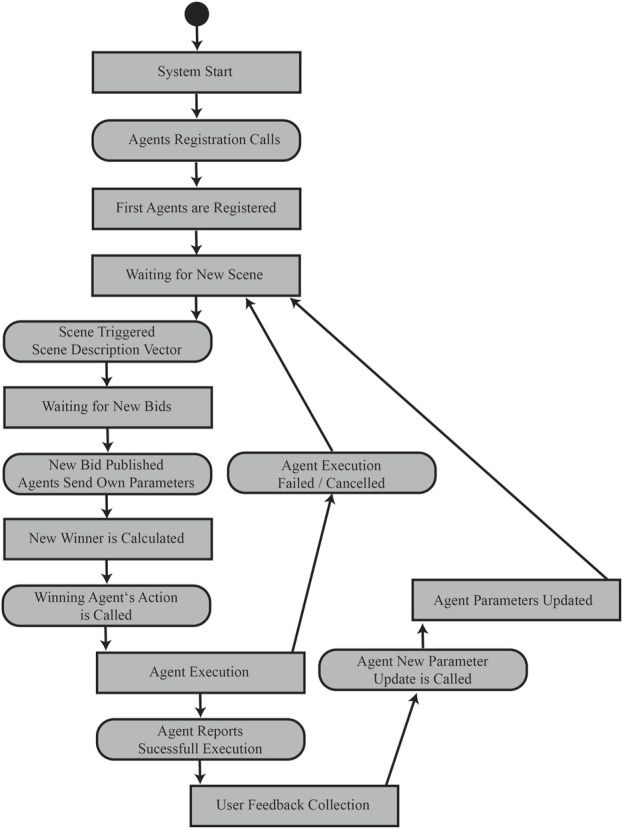
The depicted state machine shows the logic of running the architecture from an initial agent registration, a scene analysis, a bid collection and selection, the execution and feedback collection and processing.

In order to achieve simple usability in terms of the overarching objectives mentioned above, all elements of the architecture concept described above are implemented using the framework ROS. Here, the ROS2 LTS version Humble Hawksbill is used. All elements are designed either as publisher/subscriber, services or actions. The logical flow of the utilisation of the architecture is shown in a simplified state machine in Figure 3. Reflecting the structure described in Section 2.2 the elements are grouped in the four main packages Scene Analyses Layer, Marketplace Biding Platform, Agent Template and Feedback Service. Executing the architecture runs elements of the four packages in a cascaded way.

#### 2.3.1 Scene analysis layer

**FIGURE 4:**
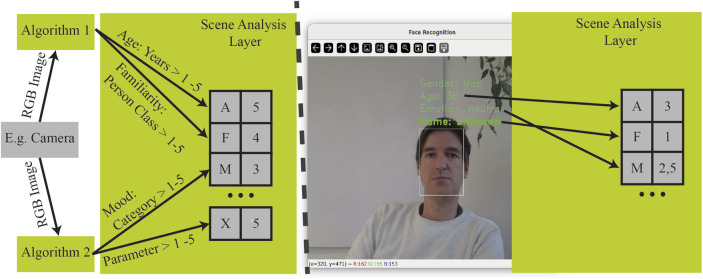
The scene analysis layer is used to collect from different information sources like algorithms pre-processing sensors data. It publishes a parameter vector describing a scene when it is triggered. On the right side an example is shown how parameters are mapped to a scene describing vector.

The purpose of the scene analysis layer is to map different information streams onto a parameter vector describing a scene. It can therefore be used to aggregate the input of multiple sensory systems and upstream algorithms. It can for instance be used to collect data from topics of a ROS instance wrapping the SHORE software (Garbas et al., 2013) and in parallel, like depicted in Figure 4 read from a ROS node using deepFace which wraps several state of the art face recognition models (Serengil und Ozpinar, 2020). Here the scene analysis layer is a subscriber of topics from both elements mentioned prior. The information derived from SHORE can be used to set a parameter “mood”. If the deepFace messages contain a name of a person a value “familiarity” can be set to five or in case of the value unknown in the name member of a deepFace message the value “familiarity” can be set to the value one. The deepFace can also be used in the described example to set a value “age”. For the experiments described in Section 3 another generic mock up scene analysis layer is used, that simply sets predefined values of an exemplary scene. Independent from subscriptions to different information acquisition topics the scene analysis layer always subscribes to a trigger topic. Once a trigger message is received an actual parameter vector filled with recently processes information from all inputs will be published. In the actual version of the described architecture this vector has a predefined number of elements, defining at the same time the set of system-wide known parameters for all agents, and the marketplace module.

#### 2.3.2 Marketplace biding platform

The marketplace biding platform receives a vector describing a scene to be handled from the scene analysis layer. This vector then represents a new bidding round on the marketplace and is filled with the parameters aggregated in the analysis layer. Receiving such a vector triggers the bidding platform to publish a topic informing all individual agents of the robot that they can hand in a new bid in form of their actual self-describing parameter vector. To stream line this process agents can register to the marketplace platform beforehand. At the moment, a unique integer value is created when agents call the registration service of the platform and the name of the agent’s ROS node name is used to identify the agents. Knowing the agents, the binding platform can close an actual biding round after it received bids from all know agents before a set deadline e.g., of 500 milliseconds is reached.

At the moment the platform then uses the Manhattan Distance, to determine the parameter set or agent closest to the current bid, since the Manhattan Distance is well suited to efficiently calculate a minimum distance even with a large number of given higher-dimensional vectors, as for example outlined by Chiu et al. (2016). After the selection based on Formula 1 the action server like described in Section 2.3.3 is called.

$$d\left(a,t\right)=\sum_{i=1}^{n}\left|p_{ai}-p_{ti}\right|$$ (1)

with: $d\left(a,t\right)$ distance between an agent and a scene

$p_{a}$ actual parameter set of an agent and a scene

$p_{t}$ parameter set describing a scene

The finals step after awaiting the execution of an agent’s action execution to finalize is the initialization of the feedback collection described in section 2.3.4.

#### 2.3.3 Agent Templates

Agents are used in the described architecture to implement and encapsulate certain characteristics of behaviour and interaction patterns. All agents carry a current and, as described in the following paragraph, continuously adaptable parameter vector with the suitability of the agent with regard to certain parameters that characterise social scenes. In the version of the architecture described here, agents are designed as subscribers of a bidding topic and as clients of a registration service in order to receive a unique integer identification number in addition to the node name, which is unique system-wide in ROS. This number can be used in future, for example, to keep several agents in a single ROS node. Parameter updates for its own characterisation can be received by means of a service offered. The actual behaviour, in which, for example, a certain form of interaction with users is mapped via short voice commands or via a graphical user interface with explanatory elements, is encapsulated in a ROS action in order to monitor the progress of an interaction and to be able to cancel ongoing interactions. For a simple implementation of new agents, all agents are derived form a template class and only the action handles are overwritten in the agent implementations.

#### 2.3.4 Feedback service

The final element completing the architecture is the feedback service. This component is triggered as a service after an agent has finished the handling of a user interaction action. In its actual implementation for experimentation a mock up feedback system can be used, to process a previously generated list of simulated user feedback automatically in individual feedback loops. Furthermore, a simple user interface can be used to manually set feedback. After retrieving the feedback either from a user interface or from a list of synthetic feedback for experimentation, the feedback instance calls a service of the respective agent to set a new vector of parameters for the agent. To calculate this new set of parameters the positive or negative distance of the user feedback from the corresponding parameter is calculated. Multiplied with a factor for the learning rate those new parameters form the next future bid of the very agent.

## 3 Results

To validate the feasibility and adaptability of the market-based behaviour selection framework, we conducted experiments, each designed to evaluate different aspects of agent performance and system response under varying conditions. In the implementation of the actual architecture especially the learning rate is of interest, as it is responsible for defining the adaption speed of the individual agents to feed back as well as the stability of the adaption process, as feedback can contain false feedback given intentionally or unintentionally.

### 3.1 Experimental parameters and initial setup

In each experiment, we defined three parameters for each agent: age, profession level, and emotional level. These parameters serve as the foundation for decision-making within the marketplace module, which uses a predefined rule set to select the optimal agent for a given task. In the following experiments the Agent 1 has an example goal parameter set of [5, 4, 1]. The initial parameters are set at [2.5, 2.5, 2.5], serving as a baseline before any feedback or adjustment is applied. Since the experiments were conducted in a simulated environment without actual robotic platforms, the selection of each agent is determined based on the rule set in the marketplace which uses the Manhattan Distance and simulated feedback.

### 3.2 Experiment 1: feasibility validation

**FIGURE 5:**
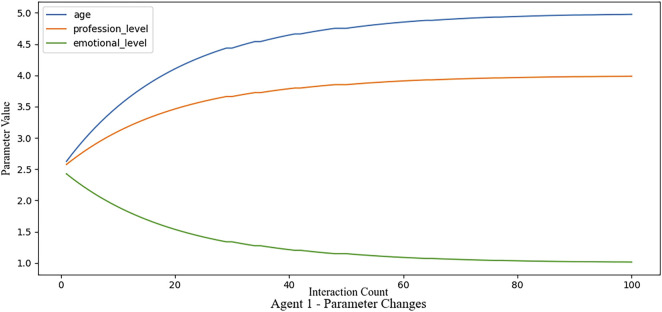
By applying constant feedback, the parameters of an agent converge to a descriptive set regarded as the best fitting values for the exemplary agent.

The first experiment aims to verify the feasibility of the framework by simulating a scenario with agents, with parameter values as defined above. In this experiment, Agent 1 is assumed to be the most suitable for the interaction scenario based on predefined rules in the marketplace. To test the adaptability of the framework, we simulate 100 feedback cycles, during which feedback is consistently provided to Agent 1. This feedback loop serves to reinforce Agent 1’s selection, allowing us to observe the framework’s ability to adjust agent parameters based on accumulated feedback as depicted in Figure 5.

### 3.3 Experiment 2: effect of feedback noise on parameter adjustment

The second experiment is designed to evaluate the system’s robustness in the presence of noisy feedback. We introduce controlled noise into the feedback loop and observe how different learning rates impact agent stability. For this experiment, we set Agent 1’s initial parameter values to [2.5, 2.5, 2.5] and assume its true parameter values to be [5, 4, 1]. The feedback loop includes three scenarios with varying learning rates.

#### 3.3.1 Conservative learning rate

**FIGURE 6:**
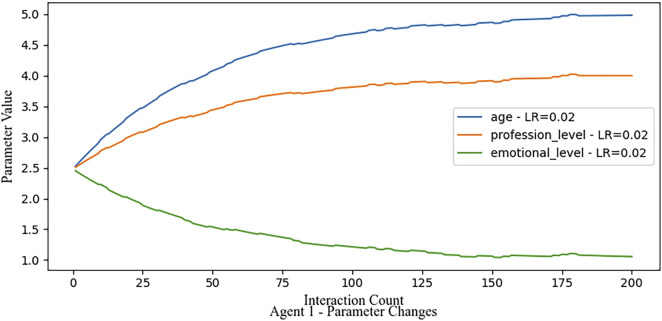
Even with noise in the feedback data a low learning rate leads to a slow but stable convergence.

With a low learning rate, we simulate 200 feedback cycles. Due to the conservative rate, parameter adjustments occur slowly, requiring a large number of cycles to reach the true values. This scenario illustrates the system’s response to slow adaptation in noisy environments like shown in Figure 6.

#### 3.3.2 Aggressive learning rate with noise

**FIGURE 7:**
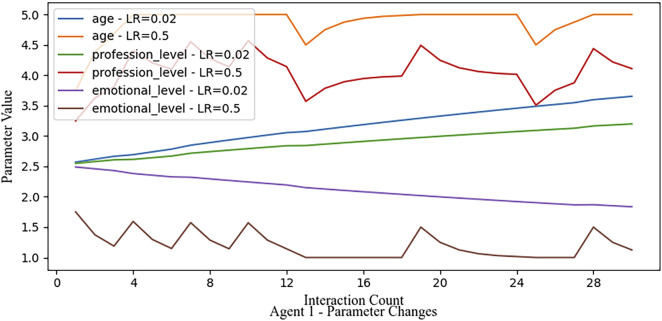
While a low learning rate of 0.02 after 30 feedback cycles only sees a directed shift of the parameter value towards the hypothetical descriptive parameter set, a more aggressive learning rate sees a conversion only after maximum of eight feedback cycles. As expected, the fast-converging rate comes at the cost of quick fluctuations when noise is introduced in the feedback.

Here, we use a high learning rate, but introduce random noise into the feedback loop, where every third feedback entry randomly assigns a value of 5. This noise causes Agent 1’s parameters to fluctuate significantly, as it quickly adjusts to feedback changes. However, when erroneous feedback (e.g., two consecutive instances of a feedback score of 5) occurs, the parameters abruptly shift to higher values, leading to instability. A comparison of the behavior in case of disturbances for a low and an aggressive learning rate is shown in Figure 7.

#### 3.3.3 Moderate learning rate

**FIGURE 8:**
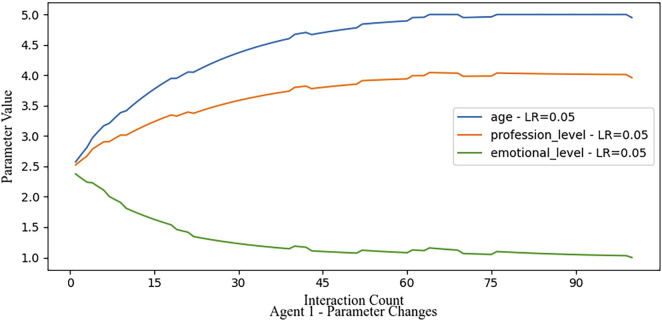
A moderate learning rate combines a medium convergence rate with rather robust reactions to noise in simulated feedback signals.

In this scenario shown in Figure 8, we apply a moderate learning rate and introduce a 5% probability of erroneous feedback. Despite the occasional noise, Agent 1’s parameters gradually converge toward a stable value near the true parameter values, demonstrating resilience to noise. This scenario exemplifies the framework’s capacity to balance stability and adaptability when the learning rate is appropriately tuned.

This experiment illustrates the importance of choosing an appropriate learning rate for stable and efficient parameter adaptation. When comparing these three scenarios, we find that a moderate learning rate strikes a balance between stability and responsiveness and maintains robustness in the presence of noisy feedback.

### 3.4 Experiment 3: automatic agent selection based on simulated user feedback

In a next experiment, the behaviour of the architecture is shown when selecting three exemplary agents. For a simple visualisation, it is assumed that the three agents are best described with the following parameters:

Agent 1: [1,1,1]
Agent 3: [3,3,3]
Agent 5: [5,5,5]

**FIGURE 9:**
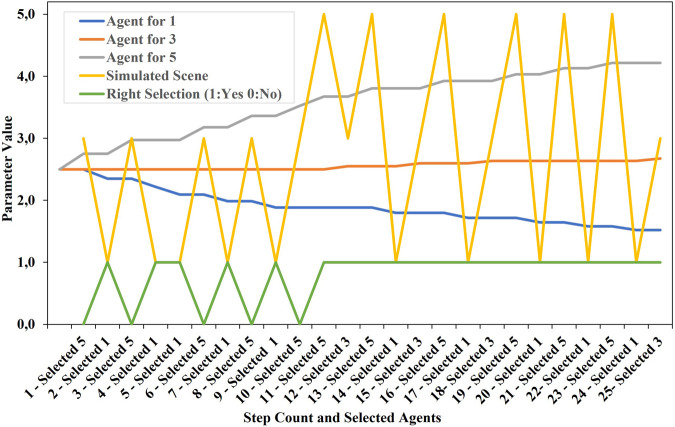
Simulated feedback for three different agents assumed as examples shows the behaviour of the architecture over 25 steps with a learning rate of 0.2. After just ten cycles, in which the agents always receive feedback corresponding to their exemplary target values, the appropriate agents are always selected in this example. The individual three parameter values of the individual agents are the same in each case.

If one of the agents is selected in a simulated experiment based on its Manhattan distance to a scene vector in a simulation step, it receives the appropriate feedback. For example, Agent 5 always receives the feedback [5,5,5]. As shown in Figure 9, the scenes, [1,1,1], [3,3,3] and [5,5,5] are alternately set as new interaction scenes on the bidding platform with a simulated scene analysis layer. The order of the scenes is chosen randomly. All agents are initially started with the values [2,5.2.5,2.5]. A factor of 0.2 is set as the learning rate for the feedback.

According to the distribution of the randomly shown scene parameters, adjustments of the individual agents in the direction of their exemplary assumed values become visible. Together with this adaptation process, an increasingly targeted selection of agents takes place, although all agents were initialised with the same parameters, as in the other examples.

## 4 Conclusion and outlook

### 4.1 Conclusion

In this contribution, we propose a new architecture for socially adaptable robots. As the goal is a rather simple to implement architecture, the ROS based approach integrates four major components scene analyses, marketplace based biding platform, agents and feedback system. The actual version of the architecture is combining an agent selection process based on the Manhattan Distance of individual agent’s descriptive parameter sets to an observed scene with a learning rate for incorporating feedback from the user that can be used to adapt the selection chances of individual agents over a number of feedback cycles. As expected, in simple simulated feedback rounds the parameters of an agent converges towards the parameter set defined as descriptive for the respective agent in the hypothetical scenarios whereas the speed of convergence is influenced by the learning rate. In addition, the feedback rate defines the stability of the parameters when statistical noise is introduced.

### 4.2 Outlook

In the presented work a simple version of the new architectural concept is introduced to describe and show the basic proof of the principle. This basic implementation leaves room for several future improvements. First, the scene analysis module at the moment is considered perfect and unfailing. To overcome this simplified solution, a scene analysis layer should also be able to deal with missing information with regard to several parameters know system wide as well as with uncertainties resulting from imperfect aggregation or upstream information processing. Either default values or a value estimation based on earlier activations of the module can be a way to compensate the aforementioned aspects. For the bidding platform the rule-based selection in this contribution is hard coded to use the Manhattan Distance of agent`s parameter sets to observed scene vectors. A modular approach for the dynamic integration and selections of rule sets and selection strategies of agents can result in a better adaptability of the architecture to different scenarios. For example, machine learning based approaches such as LLMs can be used to select different agents from a pool of multiple options when the comprehensive collection of user feedback is difficult to realise, for instance when numerous parameters form scene and agent describing vectors. Regarding the feedback collection aside from the presented approach of creating user feedback about all categories describing an agent at the end of every agent execution more sophisticated solutions are conceivable. One possible way for improvement can be a utilization of the scene analysis module when it is running in parallel to the execution of agents. For instance, a detected change of “mood” parameter of a person while interacting with an agent could be used for an automated feedback creation. Finally, the basic action behaviour of agents can be improved by rather triggering more complex state machines or behaviour trees instead of simple actions. Ultimately, state machines could replace agents in order to allow developers using the architecture to rather define scripts of interactive components and skills of a robot instead of coding individual agents for specific purposes.

**FIGURE 10:**
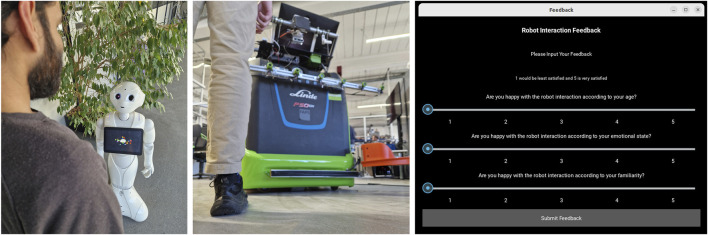
Future tests of the architecture can include robotic systems used in care or in intralogistics scenarios to collect feedback from test subjects via a graphical user interface.

The experiments presented in this article provide basic proof of the feasibility of the outlined technical solution for the adaptability of robotic systems based on feedback. Future experiments with real subjects, scenes and robot systems will be necessary to further develop and improve the proposed software architecture beyond the theoretical scenarios constructed. For example, the robot systems shown in Figure 10 can be used to obtain feedback from test subjects using a graphical user interface. Preliminary tests and workshops in care facilities, in which test subjects from different user groups had to complete simple questions and answer tasks with the robot system Pepper shown in Figure 10, indicated that care givers find voice interaction pleasant, while older residents had problems understanding the robot’s spoken output acoustically. Here, for example, practical tests can be carried out to determine whether the architecture presented adapts initially equally parameterized agents based on feedback, so that, for example, after several test runs, voice output is automatically selected for younger people and textual instructions for older people. In order to enable an investigation in a non-nursing context, studies on the navigation behavior of large transport robots can also be carried out. Here, evasive trajectories can be selected for encounters in confined spaces, for example with unknown persons. This also enables experiments on indirect feedback strategies, as generally positive or negative feedback can be mapped to all parameters of an agent for experimental purposes. In those cases, one strategy would be to shift all parameters of the agent in the direction of the recognized scene in case of positive feedback or away from the actual scene parameter set in case of negative feedback.

## Data Availability

The original contributions presented in the study are included in the article/supplementary material, further inquiries can be directed to the corresponding author. The ROS2 implementation of the architecture described in this contribution can be cloned from: https://github.com/FAU-FAPS/social_marketplace.
